# Liprin-α4 Is Required for Nickel Induced Receptor Protein Tyrosine Phosphatase-Leukocyte Antigen Related Receptor F (RPTP-LAR) Activity

**DOI:** 10.1371/journal.pone.0022764

**Published:** 2011-08-04

**Authors:** Kathrin Kiok, Hong Sun, Hailey Clancy, Sutapa Bose, Thomas Kluz, Fen Wu, Max Costa

**Affiliations:** Department of Environmental Medicine, New York University School of Medicine, Tuxedo, New York, United States of America; University of South Florida, United States of America

## Abstract

Liprin-α4 was strongly induced following nickel (II) chloride exposure in a variety of cell types including BEAS-2B, A549, BEP2D and BL41 cells. Liprin-α4, a member of the Liprin alpha family, has seven isoforms but only three of these variants were detected in BEAS-2B cells (004, 201 and 202). The level of Liprin-α4 variants 201 and 004 were highly increased in BEAS-2B cells in response to nickel. We showed that Liprin-α4 bound directly to the cytoplasmic region of RPTP-LAR (receptor protein tyrosine phosphatase-leukocyte antigen-related receptor F). The cytoplasmic region of RPTP-LAR contains two phosphatase domains but only the first domain shows activity. The second domain interacts with other proteins. The phosphatase activity was increased both following nickel treatment and also in the presence of nickel ions in cell extracts. Liprin-α4 knock-down lines with decreased expression of Liprin-α4 variants 004 and 201 exhibited greater nickel toxicity compared to controls. The RPTP-LAR phosphatase activity was only slightly increased in a Liprin-α4 knock-down line. Liprin-α4 appeared necessary for the nickel induced tyrosine phosphatase activity. The presence of Liprin-α4 and nickel increased tyrosine phosphatase activity that reduced the global levels of tyrosine phosphorylation in the cell.

## Introduction

The leucocyte common antigen-related (RPTP-LAR) protein is a member of the receptor protein tyrosine phosphatase (RPTP) F family that are single pass type I transmembrane receptor class 2A proteins [1], [2]. RPTP-LAR contains an extracellular region with three immunoglobulin-like and eight fibronectin type III domains. The cytoplasmic region of the RPTP-LAR protein includes two protein tyrosine phosphatase (PTP) domains. The first cytoplasmic domain exhibits enzymatic activity while the second domain determines specificity of substrate binding [3], [4]. RPTP-LAR plays a role in metabolic and cellular processes by its ability to respond to stimuli and transduce signals resulting in the regulation of biological processes.

Protein tyrosine phosphatase, receptor type, F polypeptide (PTPRF), interacting protein alpha 1 (PPFIA1), also known as Liprin-α1, was first identified as a binding protein of RPTP-LAR [5]. Liprin-α1 was required for the trafficking of synaptic vesicles and was involved in the development and maintenance of excitatory synapses [6], [7]. Liprin-α1 also interacted with ING4, one of five protein members of the inhibitor of growth family [8]. The ING proteins have been described as binders of histone acetylases (HAT), histone deacetylases (HDAC) and histone H3K4me3. The ING proteins are tumor suppressors of cancer invasion and metastasis [9], [10].

Liprin-α1 belongs to a family of liprin alpha proteins of which there are four members. Characteristic structure motifs shared by the family members include the N-terminal helical coil-coiled domain and three C-terminal sterile alpha motifs (SAM). The SAM domain is crucial for the direct interaction of Liprin-α1 to the second cytosolic PTP domain of the RPTP-LAR protein [11], [12]. It has been shown that phosphorylated Liprin-α1 regulates its binding to the RPTP-LAR transmembrane protein- tyrosine phosphatase. Liprin-α1 contains a C-terminal 100 amino acid sequence which is characterized as a GRIP protein binding site [13], [14]. The same sequence could be identified in Liprin-α2 and Liprin-α3 but not in Liprin-α4. A multidomain protein known as trio has also been described as an interacting partner of RPTP-LAR [15]. Liprin-α1 remains the best characterized member of the liprin alpha family. Similar to other family members, Liprin-α4 contains an N-terminal helical coil-coiled domain and three C-terminal SAM domains. Liprin-α4 belongs in this family by sequence comparison. Little is known about the cellular function of Liprin-α4. Interestingly, compared to the other three members, Liprin-α4 has the shortest N-terminal helical coiled-coil segment and it lacks the C-terminal 100-amino acid GRIP binding site found in Liprin-α1/2/3. However, the physiological function of Liprin-α4 as well as its upstream regulator and downstream targets remains largely unknown.

RNA-Seq data in A549 cells showed a more than 200-fold increase in the expression of Liprin-α4 mRNA compared to the control following a 24 h nickel exposure [15], suggesting a potential involvement of Liprin-α4 in cellular responses to nickel. Affymetrix Gene Chip array was employed to analyze the mRNA expression pattern in normal human bronchial epithelial cells (BEAS-2B) following nickel (II) chloride. An increased expression of Liprin-α4 was found. Liprin-α4 protein and mRNA levels were increased by nickel in several different cell types. In addition, nickel was able to increase the phosphatase activity of RPTP-LAR both in whole cells as well as directly using cell extracts and an *in vitro* activity assay. Interestingly, liprin-α4 knockdown cells failed to increase RPTR-LAR activity in the presence of nickel, indicating that liprin-α4 was required for nickel induced phosphatase activity. Moreover, in the absence of liprin-α4, immortalized human bronchial epithelial cells were more sensitive to nickel induced cytotoxicity. Thus, our results indicated that liprin-α4 may function as a regulator of RPTP-LAR activity and has a protective role in nickel induced cytotoxicity.

## Results

### BEAS-2B, A549, and BEP2D cells show a dose dependent increase in Liprin-α4 mRNA after 24 h exposure to NiCl2

**Figure 1:**
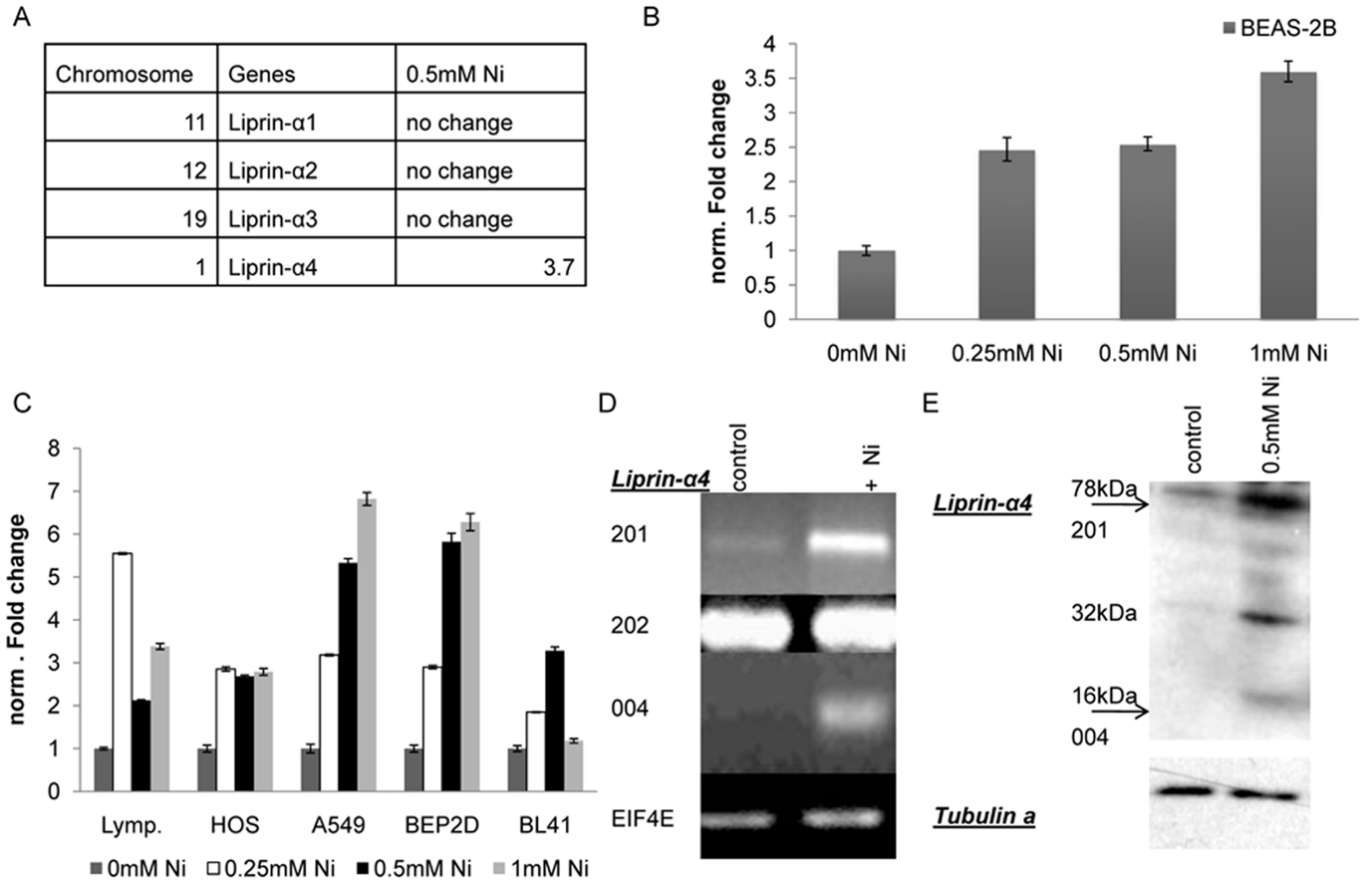
Expression of endogenous Liprin-α4 is up regulated after nickel treatment. (A) Gene Chip data showed expression for members of the liprin alpha family. Expression of Liprin-α4 was up regulated after Ni treatment but other members of the liprin family Liprin-α1/2 and Liprin-α4 were unchanged after nickel. (B) Increasing expression of Liprin-α4 in BEAS-2B cells after 24 h nickel treatment. Liprin-α4 increases in a concentration dependent manner in BEAS-2B cells. (C) Increasing expression of Liprin-α4 in different cell lines. The changes in expression were concentration dependent in A549 and BEP2D cells. (D) The isoforms 201, 202 and 004 were present in BEAS-2B cell. Liprin-α4 variant 004 and 201 was increased after nickel exposure. The isoform 202 was present in BEAS-2B cells but unchanged by nickel. (E) Western analysis shows induced Liprin-α4 protein levels for isoforms 004 (16 and 32 kDa) and 201 (78 kDa) after nickel exposure.

Affymetrix Gene Chip was used to analyze mRNA expression changes in BEAS-2B cells 24 hours following exposure to 0.5 mM NiCl_2_. 206 genes were up regulated after nickel treatment but only 66 genes were down-regulated more than two fold. Table S1 contains a list of all 272 genes that were either up or down regulated more than 2-fold following nickel exposure. We have concentrated our studies on genes that were exclusively up regulated following nickel treatment. To confirm the results of the Affymetrix chip, the expression of Liprin-α4, HMOX1, CA9 and NDRG1 were verified by quantitative PCR (Figure S1). HMOX1 showed a 3.8 fold increase after nickel treatment while Ca9 and NDRG1 increased more than 2-fold. None of the other members of the liprin alpha family (Liprin-α1, Liprin-α2 and Liprin-α3) exhibited a change in mRNA expression after nickel exposure (Figure 1A). The Liprin-α4 mRNA increased in a concentration dependent manner (Figure 1B). The Gene Chip array in BEAS-2B cells showed that Liprin-α4 was 3.7 fold up-regulated after 24 h nickel exposure (Figure 1A) and Liprin-α4 mRNA was increased after nickel treatment in comparison to control), in a concentration dependent manner (Figure 1B). Liprin-α4 was highly induced in A549, BEP2D, HOS, BL41 cells and in human lymphocytes after 24 h nickel exposure (Figure 1C). Quantitative real time PCR showed that the expression was increased in A549 and BEP2D in a concentration dependent manner. The qPCR for Liprin-α4 was performed with primers that recognized all Liprin-α4 isoforms.

### Two of seven Liprin-α4 isoforms were increased in both mRNA and protein in BEAS-2B cells exposed to NiCl2

According to the Ensembl database there are seven different Liprin-α4 protein isoforms (http://uswest.ensembl.org). We have analyzed the mRNA level of all the isoforms in untreated and nickel treated BEAS-2B cells. For this experiment we used a variant specific backward primer for reverse transcription. The expression of the isoforms was analyzed by PCR in the absence and presence of nickel. The variants 201, 202 and 004 were found to be expressed in BEAS-2B cells (Figure 1D). The Liprin-α4 isoforms 201 and 004 showed an induction in mRNA expression after nickel treatment; conversely, the expression of isoform 202 mRNA was not altered by nickel treatment. In addition to the mRNA level of Liprin-α4 we also examined the protein level of Liprin-α4 in BEAS-2B and A549 cells in the absence and presence of nickel treatment. According to Ensemble database the various Liprin-α4 isoforms resulted in different protein lengths as follows: isoform 004 contained 136 amino acids with a molecular weight of 16 kDa, isoform 201 had 692 aminoacids with a molecular weight of 78 kDa and isoform 202 contained 1186 aminoacids with a molecular weight of 134 kDa. Further analyses were performed in BEAS-2B and A549 lung cell lines but only the data from BEAS-2B cells are shown. Two out of the three isoforms were found in BEAS-2B cells based on staining with a Liprin-α4 Antibody (abcam). The Liprin-α4 isoform 004 had a molecular weight of 16 kDa (Figure 1E). In addition to this isoform a 32 kDa protein was also found which likely is the dimerized form of variant 004. As with the RNA, the protein level of cytosolic/membrane fraction of Liprin-α4 isoform 004 (16 kDa) and 201 (78 kDa) were increased 24 h after nickel exposure, as well as an increase in the possible 004 dimer of 32 kDa (Figure 1E). These two isoforms were also detected in the nuclear fraction but their expression levels in this fraction were unchanged by nickel treatment (data not shown). The protein of variant 202 was not detected despite the presence of mRNA.

### Liprin-α4 interacted directly with RPTP-LAR

**Figure 2:**
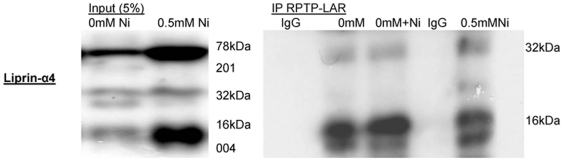
RPTP-LAR interacts with Liprin-α4. IP experiment was performed with RPTP-LAR antibody. Input shows an increasing level in Liprin-α4 after nickel treatment compared to the untreated control. Liprin-α4 binds to RPTP-LAR in absence and presence of nickel. The presence of nickel did not affect the binding efficiency between RPTP-LAR and Liprin-α4. After nickel exposure more Liprin-α4 binds to RPTP-LAR than in control. In western analysis we demonstrated an immunoprecipitation of Liprin-α4 and we showed an enrichment of Liprin-α4 that binds to the cytoplasmic region of RPTP-LAR receptor after nickel treatment.

Another member of the liprin alpha family, Liprin-α1, has been characterized as an interacting partner of RPTP-LAR. Since the mRNA level of Liprin-α1/2/3 did not change by nickel, the binding of Liprin-α4 to the cytoplasmic region of the RPTP-LAR was studied to determine if Liprin-α4 might have an effect similar to what would be expected for Liprin-α1. Immunoprecipitation (IP) using the RPTP-LAR antibody was performed to examine the direct interaction of RPTP-LAR with the Liprin-α4. The RPTP-LAR IP showed an accumulation of Liprin-α4 in control and after 24 h nickel exposure (Figure 2). We demonstrated an slightly increased binding of Liprin-α4 to RPTP-LAR in the presence of nickel. To study this binding, cell lysate were incubated, with and without nickel, to protein A/G agarose and RPTP-LAR antibody at 4°C overnight (Figure 2). Liprin-α4 was induced by nickel and there was a direct interaction between Liprin-α4 and RPTP-LAR.

### Nickel increased the RPTP-LAR phosphatase activity and reduced cellular tyrosine phosphorylation level

**Figure 3:**
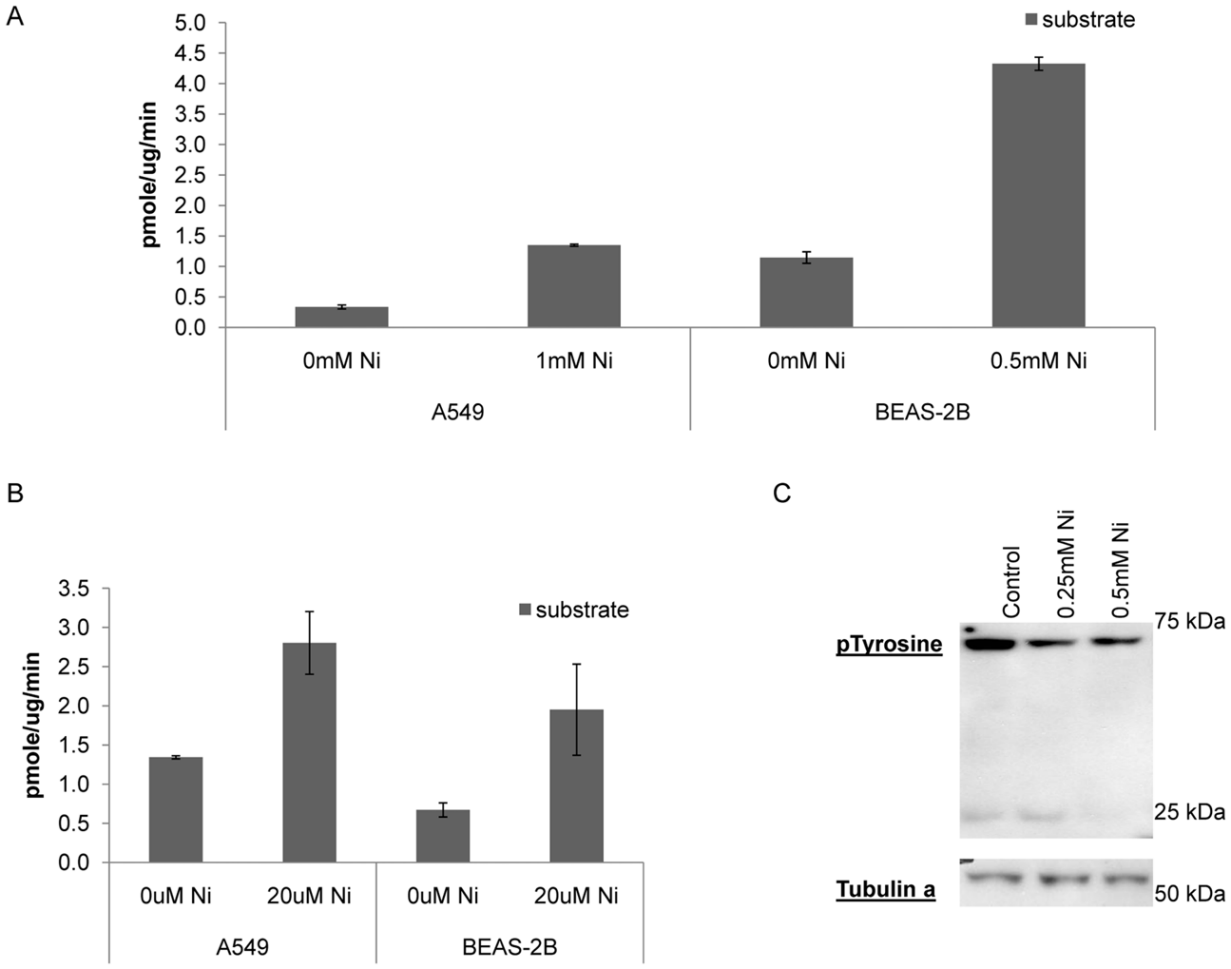
Increases protein tyrosine phosphatase activity following nickel treatment. Free phosphates which were generated by dephosphorylation reaction of the substrate were measured. (A) PTP activity was up regulated in BEAS-2B cells and A549 cells after 24 h nickel treatment. (B) Un-treated control cells showed increased phosphatase activity when nickel was added to cell extracts (C) By western analysis we demonstrated that the level of phosphorylated tyrosines in proteins was reduced in a concentration dependent manner after nickel treatment. This finding correlates with the finding that the phosphatase activity was increased after nickel exposure.

The effect of nickel on the activity of the RPTP-LAR phosphatase using a mono tyrosine phosphorylated RPTP-LAR substrate was examined by measuring the concentration of free phosphates which were generated by the dephosporylation of substrate. The phosphatase activity was measured in IP-RPTP-LAR samples in the absence and presence of nickel. The reaction in the absence of substrate was utilized as background and these values were subtracted from each sample. The RPTP-LAR phosphatase showed a higher activity in nickel treated cells compared to both the control without enzyme and in the untreated BEAS-2B and A549 cells (Figure 3A). We used the monophosphorylated substrate (PTP Substrate 1-pTyr) from the Insulin Receptor which was recognized by RPTP-LAR. In A549 cells the activity increased from 0.3 in control to 1.35 pmole/ug/min after a 24 hrs 1 mM nickel treatment (non-toxic dose of Ni, Figure 3A). There was a similar increase in BEAS-2B cells, from 1.15 to 4.33 pmole/ug/min after 0.5 mM nickel exposure (non-toxic dose of nickel). In order to compare equally toxic nickel doses we exposed the two cell lines to different nickel concentrations, since BEAS-2B cells were twice as sensitive to nickel compared to A549 cells. Additionally the activity of the membrane bound RPTP-LAR tyrosine phosphatase was analyzed in the presence and absence of nickel in BEAS-2B and A549 cells using a tyrosine phosphatase assay (Figure 3B). The tyrosine phosphatase was more active in the presence of nickel. The presence of 20 uM nickel in the reaction led to an increased activity from 1.34 to 2.8 pmole/ug/min in A549 and we also found an increase from 0.7 to 1.95 pmole/ug/min in BEAS-2B cells (Figure 3B). An increased phosphatase activity was also observed in the lung cell line BEP2D and in a bone cancer cell line HOS 24 h following nickel exposure (Figure S2). The tyrosine phosphorylation level of proteins after nickel exposure was examined using western blot. Decreased tyrosine phosphorylation levels in two cytoplasmic proteins were found after nickel treatment by western blot (Figure 3C). The results in figure 3 represented multiple repeated experiments.

### Liprin-α4 knockdown increases the cellular toxicity to nickel and it attenuates the nickel induced increased phosphatase

**Figure 4:**
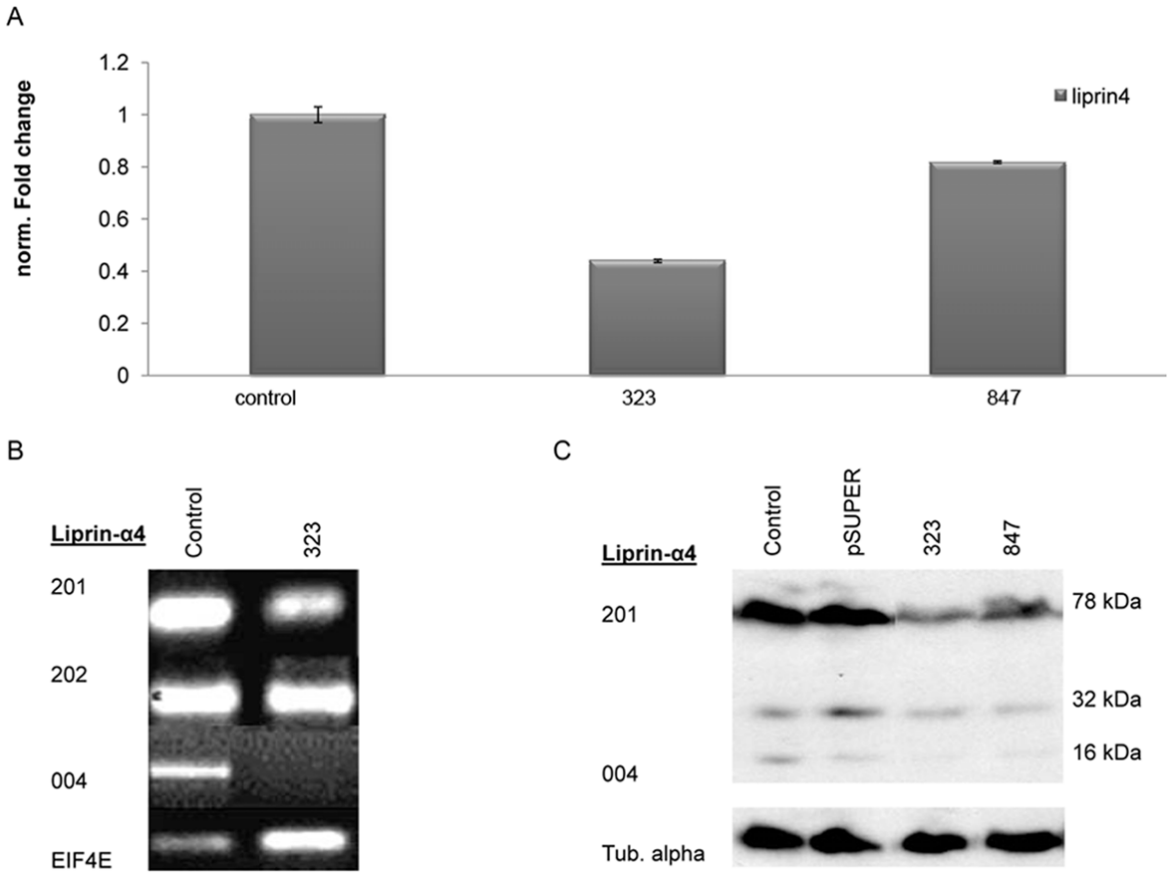
Expression of Liprin-α4 in RNAi lines. The control pSUPER contains the vector without the RNAi construct and knock-down lines contain the vector including the siRNA construct against Liprin-α4. (A) Liprin-α4 is 50% down regulated in 323 and it is decreased approximately 20% in RNAi line 847. (B) Expression of isoform 004 is not detectable in RNAi line 323. The variant 201 is decreased in the knock-down 323 line. The amount of Liprin-α4-202 is not changed in 323. (C) In Western analysis the RNAi lines show a reduced amount of Liprin-α4 isoform 004 (16 kDa) and isoform 201 (78 kDa) in both knockdown lines (323 and 847). The isoform 201 shows a slight decrease in RNAi line 323.

**Figure 5:**
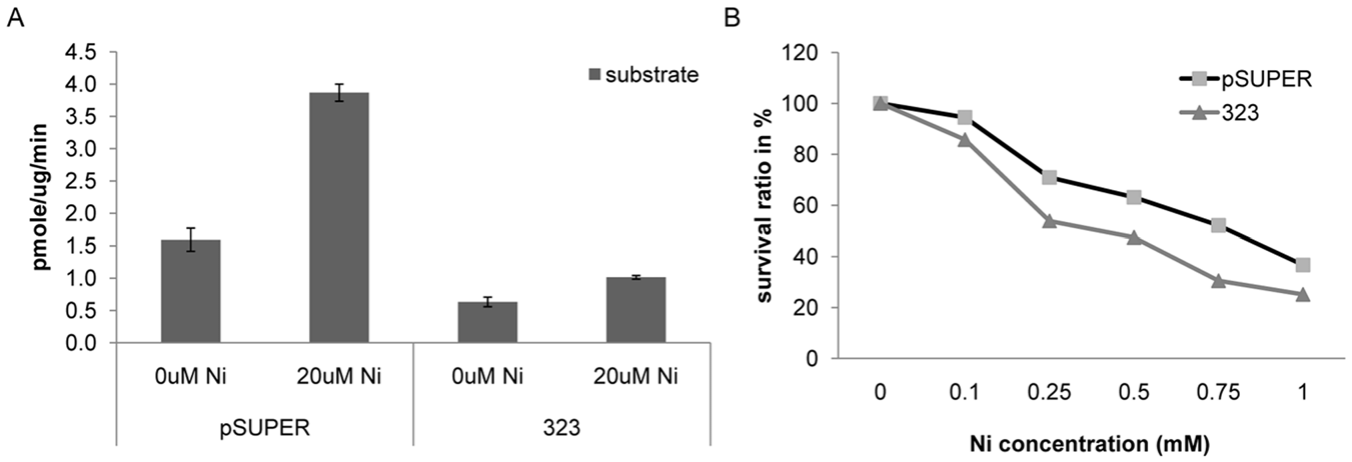
Cellular effects of Liprin-α4 knock-down in BEAS-2B cells. (A) In RNAi line 323 the PTP activity was slightly increased compared to control. (B) Liprin-α4 RNAi line 323 showed a higher sensitivity to nickel induced cytotoxicity compared to the control.

Two stable Liprin-α4 RNAi BEAS-2B cell lines were established. The transcription of Liprin-α4 in both cell lines was analyzed using quantitative PCR. In line 847 Liprin-α4 was expressed at 80% of the RNA level of the control, while in cell line 323 the expression of Liprin-α4 was reduced to 50% (Figure 4A). We showed that in the knock-down cell line 323, as compared to the control, the variant 201 was reduced in expression, the variant Liprin-α4 004 was not detectable by qPCR while the isoform 202 remained unchanged (Figure 4B). The protein level of Liprin-α4 was examined in the knock-down lines 323 and 847 (Figure 4C). Western blot analysis reveals that the isoform 004 was reduced in both of the RNAi lines, while the protein variant 201 (78 kDa) only showed a decreased level of expression in Liprin-α4 knock-down line 323. The Liprin-α4 isoform 202 protein was not detected in this line. It was possible that the variant 202 was not translated into protein or that the Liprin-α4 antibody did not recognize this isoform. We investigated the phosphatase activity in the presence of nickel in both the control and in the knock-down line 323 (Figure 5A). The control showed an increasing activity from 1.6 to 3.9 pmole/ug/min in the presence of nickel while in the knock-down line 323, where Liprin-α4 was reduced by 50%, the activity increased much less (0.6 to 1.0 pmole/ug/min). These results showed that Liprin-α4 was necessary for the nickel-induced activity of phosphatase. These results indicated that the variants 004 and 201 played a role in the cellular response to nickel. The RNAi line was treated with various nickel concentrations for 24 h and colony formation assay was performed (Figure 5B). Knock-down of Liprin-α4 led to an increased sensitivity to nickel toxicity in BEAS-2B (Figure 5B) and in A549 cell lines (data not shown). For example, the survival rate for the control was 70% and it was reduced to 53.8% in the knock-down line 323 after 0.25 mM nickel treatment for 24 h, demonstrating a significantly higher sensitivity of line 323 in comparison to control (Figure 5B).

### Liprin-α4 was able to bind to nickel

**Figure 6:**
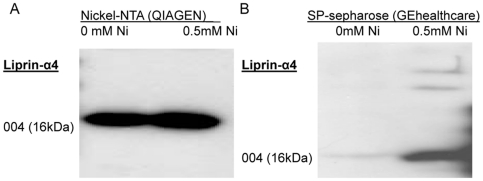
Liprin-α4 binds directly to Ni-NTA column. (A) Liprin-α4 binds directly to nickel-NTA agarose. (B) SP-Sepharose was pre-incubated with nickel. Ni-sepharose is able to bind Liprin-α4.

Increased RNA and protein levels of Liprin-α4 were found after nickel treatment and the knock-down line showed greater sensitivity to nickel than did the control. In addition the increased phosphatase activity in the presence of nickel was dependent on Liprin-α4. Next, the direct binding of nickel to Liprin-α4 and/or to the cytoplasmic region of the receptor RPTP-LAR was examined. Ni-NTA agarose was used to study the direct binding of Liprin-α4 to nickel. It was known that Ni-NTA bound histidine tagged proteins and was generally used for their purification. To the best of our knowledge this experiment is the first time that Ni-NTA agarose was used to study a specific protein binding to nickel. Briefly, Ni-NTA and lysate were incubated over night. Following this incubation, the Ni-NTA agarose was washed four times with lysis buffer. The agarose interacting proteins were analyzed by gel electrophoresis and incubated with the Liprin-α4 antibody (Figure 6A). The direct binding of Liprin-α4 to nickel was shown in the cytoplasmic protein extract by overnight exposure to Ni-NTA agarose (Qiagen). This experiment suggested a direct interaction between Liprin-α4 and nickel (Figure 6A) but without a negative control the binding specificity was questionable. We incubated SP-sepharose with nickel over night. After nickel treatment the sepharose was incubated with cell lysate at 4°C over night. Non specific sepharose protein binding was eliminated by washing the sepharose four times with lysis buffer. The cleaned sepharose samples were diluted in sample buffer and separated in protein gel. The membrane was stained with the Liprin-α4 antibody. The direct binding of Liprin-α4 to nickel treated sepharose was confirmed (Figure 6B) but there was no direct binding of RPTP-LAR to nickel with Ni-NTA or with Ni treated sepharose. Liprin-α4 was a crucial player in the response of BEAS-2B cells to nickel. These results demonstrate that Liprin-α4 was a direct binding partner of the receptor protein tyrosine phosphatase RPTP-LAR and nickel.

## Discussion

In this study Liprin-α4 expression was increased following nickel treatment. Recently Mattauch et al. [16] had described an up-regulation of Liprin-α4 in response to hypoxia. Its activation occurred by binding of the hypoxia-inducible factor 1A (HIF-1A) to HRE consensus binding site in the promoter region of Liprin-α4. There are common mechanisms between nickel and hypoxia in particular HIF-1A is stabilized by both nickel and hypoxia treatment [17], [18]. Nickel has been shown to inhibit the Prolyl-hydroxylase that was responsible for marking HIF-1A for degradation and nickel exposed cells exhibit hypoxia signaling under normal oxygen. Following nickel exposure, Liprin-α4 was up-regulated by the binding of HIF-1A to the HRE in the Liprin-α4 promoter. Besides Ni(II) also Co(II) and to al lesser Cu(II), Zn(II), As(III), Mn(II) and V(V) lead to a stabilization of HIF-1A protein by inhibition of its hydroxylation [19] and this would induce Liprin- α4 because it has an HRE in its promoter. Busch et al. (2010) reported an increasing expression of Liprin- α4 after 3 h treatment with Co [20]. Other metals such as Al(III) and Cd(II) do not induce HIF-1A. It was shown that Chromium Cr(VI) stabilized HIF-1A protein at a highly toxic doses after 4 h exposure in A549 cells whereas after 24 h exposure in A549 to chromium the protein HIF1A was not detected. We were unable to measure any significant changes in Liprin-α4 expression after 24 h chromium treatment (Wu, data not published). The effect of other metals on Liprin-α4 expression remains unclear.

Ni-induced Liprin-α4 expression seems to be due to the stabilized HIF-1A protein which binds to HRE consensus site in the Liprin-α4 promoter and activates its expression. Furthermore Liprin-α4 did not show a significant change in expression in Beas-2B cells transformed by long-term exposure to nickel (Clancy, data not published). This additional information seems to indicate a direct function of Liprin-α4 in nickel-induced toxicity but not in carcinogenicity since it is able to bind nickel ion. It is known that several metal ions like nickel, cobalt and chromium induced oxidative stress after cell treatment. Salnikov et al. (2000) showed that metals induced the production of reactive oxygen species (ROS) by activating hypoxia signaling under normal oxygen tension. This formation of ROS is separate from the activation of HIF-1A dependent genes including Liprin-α4 [19]. This information indicates that stabilization of HIF-1A induces the overexpression of Liprin- α4. Besides HIF-1A dependent induction of Liprin- α4 we have shown a transcriptional activation of the stress-inducible gene HMOX1. This protective mechanism is mediated by transcription factor nuclear erythroid 2-related factor2 (NRF2) that can regulate expression of HMOX1 and other antioxidants [21]. Liprin-α4 seems to play a role in cellular protection against nickel-induced toxicity. The oxidative stress has not been ruled out by the data presented here, but induction of HIF-1a appears to be the main mechanism by which Ni induces Liprin-a4 expression. The up-regulation of Liprin-α4 in response to nickel was most likely involved in attenuating the toxicity of nickel since there was an increased sensitivity to nickel in a Liprin-α4 knock-down cell line. Future investigations should include overexpression of Liprin-α4 to analyze its potential of protection against nickel-induced toxicity.

Liprin-α4 is a member of the liprin family that contains an N-terminal coiled-coil domain and the C-terminal domain includes three sterile alpha motifs. The most studied member of the liprin alpha family is Liprin-α1, which is an interaction partner of GIT, KIF1A, ERC, EMS and GRIP [22], [23], [24], [25]. Initially, Liprin-α1 was described as an interacting partner for receptor protein tyrosine phosphatase RPTP-LAR that binds directly to the second phosphatase domain of RPTP-LAR family members [23]. Besides Liprin-α1, the multidomain protein trio also binds to the LAR transmembrane tyrosine phosphatase [5], [22]. This interaction between Liprin-α1 is regulated by autophosphorylation on a serine residue [2]. By sequence homology Liprin-α2/3 and 4 belong to this family. Here we showed a direct interaction of Liprin-α4 with RPTP-LAR receptor protein that led to an increased activity of this phosphatase. An increase in activity of this phosphatase was found after nickel treatment and in the presence of nickel. In the absence of Liprin-α4, the phosphatase activity is not significantly increased by nickel. This suggested a direct connection between Liprin-α4, RPTP-LAR and nickel. So far we have identified an interaction between Liprin-α4 and the RPTP-LAR protein and between Liprin-α4 and nickel but not between RPTP-LAR and nickel. It seems that nickel enhanced the binding of Liprin-α4 to RPTP-LAR. Liprin-α4 and nickel were necessary for the increased phosphatase activity. The binding of Liprin-α4 to RPTP-LAR did not appear to be regulated by its phosphorylation. In contrast, the tyrosine phosphorylation level was decreased.

**Figure 7:**
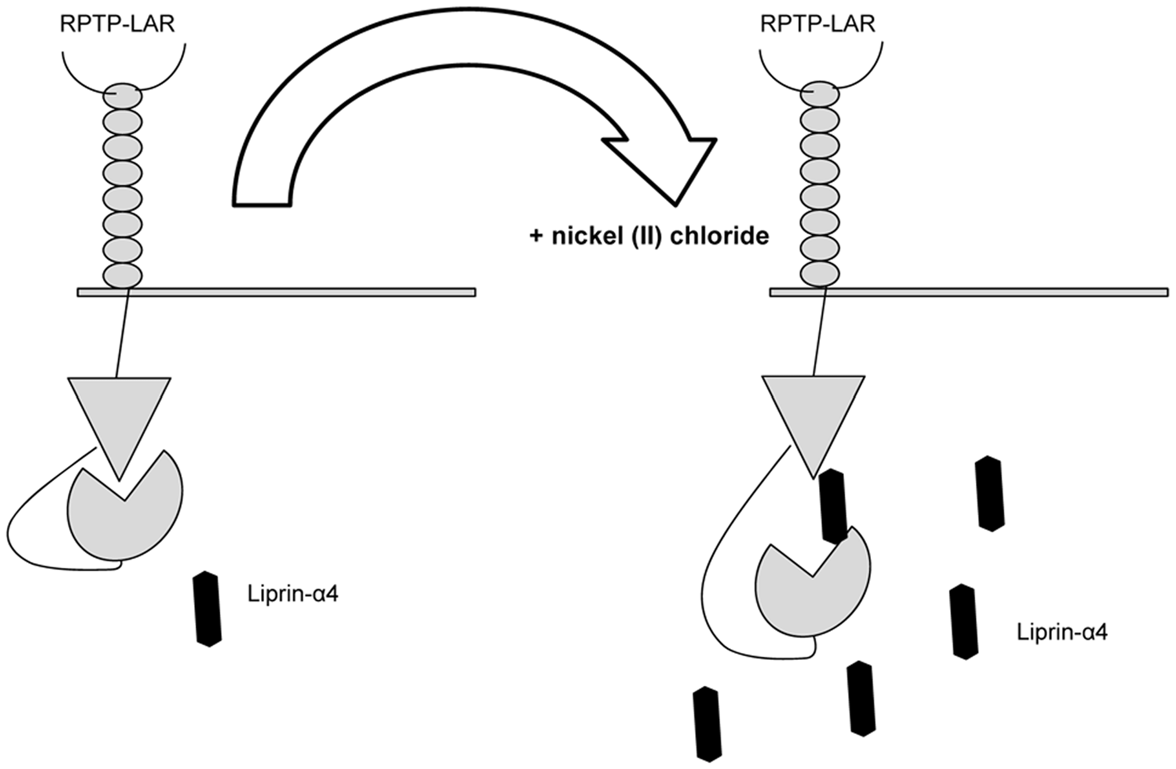
Increasing phosphatase activity in presence of nickel and Liprin-α4. The activity of RPTP-LAR remained at a basal activity level in the control and in Liprin-α4 knock-down line 323. In the presence of nickel, Liprin-α4 binds to the second phosphatase domain (D2) of RPTP-LAR. By reorganization of RPTP-LAR, the first phosphatase domain (D1) might undergo conformational arrangements and become more open for its phosphate substrates.

We have developed a model for Liprin-α4 and its role in the cellular response to nickel (Figure 7). The activity of RPTP-LAR remained at a basal activity level in the control and in Liprin-α4 knock-down line 323. In the presence of nickel, Liprin-α4 binds to the second phosphatase domain of RPTP-LAR which is similar to the interaction between RPTP-LAR and Liprin-α1. This interaction could lead to a structural rearrangement of the first phosphatase domain. Only the first domain of the RPTP-LAR protein shows phosphatase activity. By reorganization of RPTP-LAR, the first phosphatase domain might undergo conformational arrangements and become more open for its phosphatase substrates.

The increasing activity of RPTP-LAR by Liprin- α4 seems to play a special role in response to nickel. PTPs effect a number of cell processes including cell growth, differentiation and the mitotic cycle. The altered expression of Liprin- α4 and its resulting increased RPTP-LAR activity may be related to a nickel- induced stress response with as yet undetermined downstream effects.

## Materials and Methods

### Cell culture

HOS [26]and BEAS-2B [27]cells were grown in Dulbecco's Eagle's medium (DMEM). BEP2D [28] cells were cultured in LHC8 medium (GIBCO). BL41 [29] and Lymphocytes were cultured in RPMI 1640 medium (Invitrogen). A549 [30] were maintained in F-12K medium (Invitrogen). 10% fetal bovine serum (FBS, ATLANTA biochemical's) and 1% penicillin/streptomycin (GIBCO) were added to all media. The cell lines were cultured at 37°C in a humidified atmosphere of 5% CO_2_. Cells were exposed to different concentrations of nickel (II) chloride for 24 h. Transfected cells were treated with 0.1 uM puromycin.

### Colony formation assay

Cells were seeded at a density that yielded 80% confluence. The following day, cells were exposed to various nickel concentrations for 24 hours; cells were then trypsinized and counted and 300 cells were seeded per 100 mm dish. After 2 weeks, media were removed, dishes washed twice with cold PBS and stained with Giemsa reagent for 30 min. Dishes were rinsed with water and dried before manual counting.

### Affymetrix Gene Chip expression

Total RNA was isolated from control and treated samples using Trizol (Invitrogen) according to the manufacturer's protocol. Samples were purified with the RNaesy kit (QIAGEN). cDNA probes were synthesized and labeled using Gene Chip Whole Transcript cDNA Synthesis and Amplification Kit and Terminal Labeling Kit (Affymetrix), and subjected to hybridization with Gene Chip Human Gene 1.0 ST Array (Affymetrix) that contained 28,869 well annotated genes. Hybridization and scanning of the arrays was performed using a standard procedure. Microarray data was analyzed using GeneSpring v11 (Agilent Technologies). The expression value of each probe set was determined after quantile normalization using RMA16 algorithm and baseline transformation to the median levels of all samples.

### Antibodies

The following antibodies were obtained from commercial suppliers: Monoclonal mouse anti-Liprin-α4 (ab56339), Abcam and Monoclonal mouse alpha Tubulin (T9026) Sigma-Aldrich. The antibodies p-Tyrosine (PY350), RPTP-LAR (R-20): sc1119, anti-goat IgG-HRP (sc-2384), anti- mouse IgG-HRP, normal IgG (sc-2028) and Protein A/G Plus-agarose (sc2003) were purchased from Santa Cruz.

### RNAi interference and transfection

Short interfering RNA for Liprin-α4 was cloned to form a hairpin structure in vector pSUPER (Oligoengine) according to the manual. The sequences of the siRNA against Liprin-α4 were as follows: Liprin-α4-847 5′-GCAGCTGGATGCCATCAAT- 3′ and Liprin-α4-323 5′ CCATTAGAAGATCCTTCTT- 3′. Exactly 1 ug of vector with and without RNAi construct was transfected into BEAS-2B and A549 cells according to the Lipofectamine Plus (Invitrogen) protocol.

### RNA isolation and RT-PCR

RNA was isolated with Trizol following the manufacturers protocol. RNA was purified with 8 M LiCl for 2 h on ice and precipitated with 1/10 volume NaAc, followed by 2.5 times the volume of 100% ethanol over night at −20°C. The purified RNA was used for RT-PCR with random primer and Liprin-α4 isoform specific reverse primer with Superscript III (Invitrogen). The isoform specific PCR was performed with Maxima Hot Start polymerase (Fermentas) with 58°C annealing and one min at 72°C. The following primers were used: F1- GAGCTGGCCCAGAGAATTGCAGC, **R1-** TGGTGCTGCTCCTCAATCTGCCG, F1b- GAGATCG GCATCAGCAATGCCCTG, **1b-** CCATTCATTCCCAATCCACTCATG, F2- GCGTCTG CAGCTCCACCTGAAGG, F4- GCCGTTTGTGGATGGCGTCCAC and **R2-** CAATGCCGAG TAGCGGCGATGCAC. The quantitative PCR was conducted using ABI 7300 detection system with Power Sybr Green (ABI). The following primers were used for normalization EIF4EBP2F7139 AGAGGACAC TCACGTGCTAAGGT, EIF4EBP2R7274 CAGTCTCCGCTTTTCCATTCA and for the genes of interest Liprin-α4F974 CAGCATGGAAGCCCTAAACCT, Liprin-α4R1076 GGA CAGGGCCGTCAGAGA, CA9F1284rt CATCCTAGCCCTGGTTTTTGG, CA9-R1365rt CCTTCTG TGCTGCCTTCTCAT, HMOX1 F1212 GCCCTTCAGCATCCTCAGTTC, HMOX1R1280 GGTTTGAGACAGCTGCCACAT, NDRG1F654rt TACCGCCAGCACATTGTGAAT and NDRG1 R723rt GGCTGTTGTAGGCATTGATGAA in different cell lines after nickel exposure. For quantitative Real time PCR the primers were designed with primer express 3.0 (ABI).

### Protein isolation, Western

Cells were cultured in 150 mm dishes, washed twice with PBS and once with PBS/1 mM EDTA. Cells were scraped in PBS/1 mM EDTA. The cells were resuspended in buffer (10 mM HEPES pH 7.9, 50 mM NaCl, 0.5 M Sucrose, 0.1 mM EDTA, 0.5% Triton X100, freshly added 1 mM DTT, 1 mM PMSF, Roche proteinase inhibitor). After incubation on ice the suspension (cytoplasmic/membrane fraction) was placed in a new tube. This fraction was used for Western analysis. The pellet (nuclear fraction) was washed with buffer A (10 mM HEPES pH 7.9, 10 mM KCl, 0.1 mM EDTA, 0.1 mM EGTA, freshly added 1 mM DTT, 1 mM PMSF, Roche proteinase inhibitor). The nuclear extract was diluted in buffer 2×C (10 mM HEPES pH 7.9, 500 mM NaCl, 0.1 mM EDTA, 0.1 mM EGTA, 0.1% IGEPAL add 1 mM DTT, 1 mM PMSF, Roche proteinase inhibitor). The protein concentration was determined using the Bio-Rad protein assay and 50 ug protein from cytoplasmic/membrane fractions were separated by 10% SDS-PAGE gel and transferred to polyvinylidene difluoride (PVDF) membranes. Immunoblotting was performed using antibodies Tubulin, Liprin-α4, RPTP-LAR and p-Tyrosine. Concentrations of the antibodies were utilized depending upon the recommendations of the suppliers.

### Immunoprecipitation

Cells were lysed with RIPA buffer for the immunoprecipitation (IP) experiment. Control IgG was used to remove non specific binding proteins. The IP experiment was performed with RPTP-LAR antibody or Liprin-α4 or pTyrosine antibody according to the Santa Cruz protocol. The precleaned lysate was also incubated with Ni-NTA agarose (QIAGEN) and to SP-Sepharose Fast Flow (GE Healthcare, 17-0729-10) in the presence nickel (II) chloride (SIGMA) overnight at 4°C. The IP samples were washed four times with RIPA buffer. Proteins were loaded on 8%, 10% or 12% acrylamide gels. Concentrations of the antibodies were utilized depending upon the recommendations of the suppliers.

### PTP assay

Cells were maintained in 150 mm dishes, washed twice with 0.9% NaCl and diluted in lysis buffer (50 mM HEPES pH 7.4, 0.5% Triton X100, 10% Glycerol, Protease inhibitor Roche, 1 mM PMSF). Control IgG was used to remove non specific binding proteins. The precleaned cell lysate was incubated with RPTP-LAR antibody and Protein A overnight at 4°C. The IP samples were washed four times with lysis buffer and eluated with 10 mM Imidazol. The protein concentration was determined using the Bio-Rad DC protein assay, and 50 ug proteins were separated by 10% SDS-PAGE gel. The tyrosine phosphatase assay was performed according to the instruction of the “protein tyrosine phosphatase assays kit” (Sigma-Aldrich). We used Monophosphate Substrate (PTP Substrate 1) [pTyr1146] Insulin Receptor (1142–1153) as substrate for RPTP-LAR. Experiment was repeated several times.

Cells were diluted in lysis buffer (50 mM HEPES pH 7.4, 0.5% Triton X100, 10% Glycerol, Protease inhibitor Roche, 1 mM PMSF). The PTP assay was perfomed with the lysate according to the instruction of the “protein tyrosine phosphatase assays kit” (Sigma-Aldrich, Figure S2). We got reproducible results.

## Supporting Information

Figure S1 Validation of Affymetrix GeneChip data from BEAS-2B cells exposed to NiCl_2_ with quantitative real time PCR. We found an increased expression of Liprin-α4, CA9, NDRG1 and HMOX1 after nickel treatment. These four genes remain unchanged in control.
(TIF)

Figure S2 Phosphatase activity is increased after Nickel treatment in a concentration dependent manner in BEP2D and HOS cells.
(TIF)

Table S1 Complete list of 272 genes differentially expressed more than 2-fold after nickel exposure.
(PDF)
