# Supplementary figures and images for: Liprin-α4 Is Required for Nickel Induced Receptor Protein Tyrosine Phosphatase-Leukocyte Antigen Related Receptor F (RPTP-LAR) Activity
Source: PLoS One. 2011 Aug 4;6(8):e22764. doi: 10.1371/journal.pone.0022764 (PMC3150438; doi:10.1371/journal.pone.0022764)

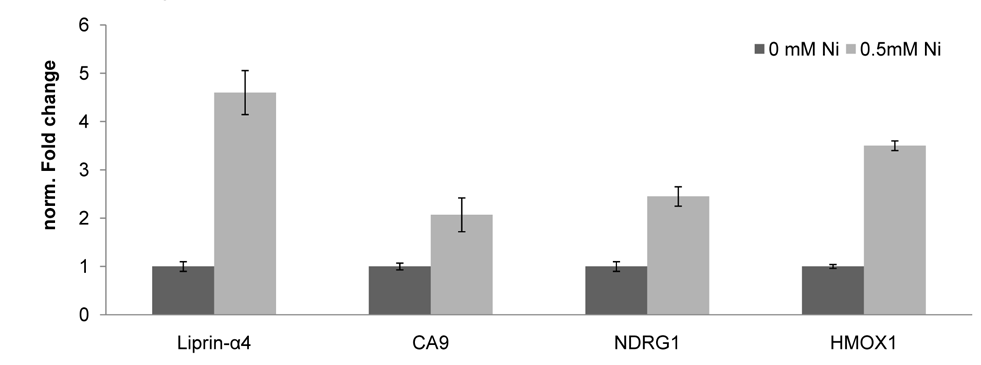

Supplement: Figure S1 — Validation of Affymetrix GeneChip data from BEAS-2B cells exposed to NiCl2 with quantitative real time PCR. We found an increased expression of Liprin-α4, CA9, NDRG1 and HMOX1 after nickel treatment. These four genes remain unchanged in control. (TIF) [file pone.0022764.s001.tif]

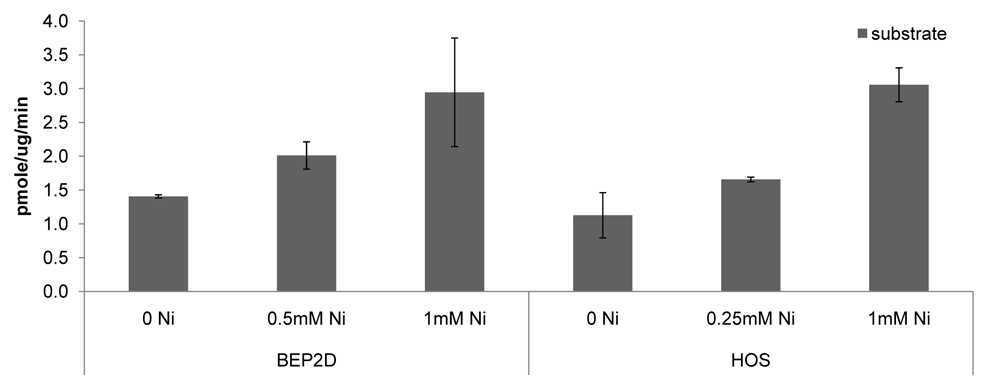

Supplement: Figure S2 — Phosphatase activity is increased after Nickel treatment in a concentration dependent manner in BEP2D and HOS cells. (TIF) [file pone.0022764.s002.tif]
