# Supplementary material for: Liprin-α4 Is Required for Nickel Induced Receptor Protein Tyrosine Phosphatase-Leukocyte Antigen Related Receptor F (RPTP-LAR) Activity
Source: PLoS One. 2011 Aug 4;6(8):e22764. doi: 10.1371/journal.pone.0022764 (PMC3150438; doi:10.1371/journal.pone.0022764)
Supplement: Table S1 — Complete list of 272 genes differentially expressed more than 2-fold after nickel exposure. (PDF) [file pone.0022764.s003.pdf]

Supplemental Table 1. Complete list of 272 genes differentially expressed more than 2-fold after nickel exposure

Affymetrix ID	Gene Symbol	Ni vs control	Regulation Ni
8153002	NDRG1	**10.28**	up
8155083	CA9	**10.22**	up
7928308	DDIT4	**9.59**	up
8081288	TMEM45A	**9.20**	up
7961891	BHLHB3	**8.85**	up
8135915	HIG2	**8.21**	up
8086961	PFKFB4	**7.97**	up
7926037	PFKFB3	**7.14**	up
8004408	FGF11	**5.99**	up
8122265	TNFAIP3	**5.99**	up
8004360	KCTD11	**5.89**	up
7989365	RORA	**5.32**	up
8136940	FAM139A	**4.76**	up
8119898	VEGFA	**4.64**	up
8113709	LOX	**4.63**	up
8083494	MME	**4.52**	up
7909104	PCTK3	**4.50**	up
8062190	SPAG4	**4.15**	up
8160297	ADFP	**4.13**	up
8150186	RNF122	**4.11**	up
8136954	FAM139B	**4.09**	up
8065948	FER1L4	**4.07**	up
7946641	GALNTL4	**4.00**	up
8064790	RASSF2	**3.87**	up
8072678	HMOX1	**3.85**	up
8038261	GYS1	**3.84**	up
8055465	CXCR4	**3.82**	up
7900540	FAM80A	**3.74**	up
8049825	HDLBP	**3.73**	up
8083223	C3orf58	**3.71**	up
7953532	ENO2	**3.69**	up
8097282	SPRY1	**3.69**	up
7908879	PPFIA4	**3.69**	up
7920642	MUC1	**3.64**	up
7995292	SLC6A8	**3.63**	up
8111932	CCL28	**3.61**	up
8048489	ANKZF1	**3.60**	up
7912157	ERRFI1	**3.60**	up
8086517	CDCP1	**3.60**	up
8128123	RRAGD	**3.54**	up
8029219	TMEM145	**3.51**	up
8077441	BHLHB2	**3.51**	up
8115851	STC2	**3.50**	up
8149629	GFRA2	**3.47**	up
7987248	GOLGA8A	**3.46**	up
8059111	ABCB6	**3.40**	up
7987279	GOLGA8B	**3.37**	up
8083594	PTX3	**3.32**	up
8105191	PARP8	**3.30**	up
8113981	P4HA2	**3.28**	up
7973084	ANG	**3.27**	up
7985587	SCAND2	**3.21**	up

Affymetrix ID	Gene Symbol	Ni vs control	Regulation Ni
7934278	P4HA1	**3.18**	up
8112841	HOMER1	**3.14**	up
7943413	BIRC3	**3.14**	up
8131067	GPR146	**3.13**	up
8046408	PDK1	**3.13**	up
8056837	GPR155	**3.10**	up
7934997	PPP1R3C	**3.06**	up
7962183	AK3L1	**3.02**	up
7937079	BNIP3	**3.00**	up
8013660	ALDOC	**3.00**	up
8162940	ABCA1	**2.99**	up
8129985	PLAGL1	**2.96**	up
7965423	BTG1	**2.96**	up
8043283	JMJD1A	**2.91**	up
8060745	SMOX	**2.90**	up
8089596	WDR52	**2.83**	up
8085033	LMLN	**2.82**	up
8020847	DTNA	**2.79**	up
8042942	HK2	**2.78**	up
7964460	DDIT3	**2.76**	up
8093053	TFRC	**2.73**	up
8154100	VLDLR	**2.72**	up
8024170	HMHA1	**2.72**	up
8083839	GPR160	**2.71**	up
7956631	FAM119B	**2.71**	up
8064766	RNF24	**2.71**	up
8166511	PDK3	**2.69**	up
8116096	DDX41	**2.68**	up
7915472	SLC2A1	**2.66**	up
8044766	INSIG2	**2.66**	up
7980828	CCDC88C	**2.65**	up
8053484	ST3GAL5	**2.64**	up
7905664	SLC27A3	**2.64**	up
8149825	STC1	**2.64**	up
8114845	PRELID2	**2.64**	up
8169263	VSIG1	**2.62**	up
8047565	ALS2CR13	**2.60**	up
8075483	PIK3IP1	**2.59**	up
8166714	LANCL3	**2.59**	up
7971015	SMAD9	**2.59**	up
8175016	APLN	**2.59**	up
7927876	TET1	**2.58**	up
8166184	CA5B	**2.57**	up
8127778	FAM46A	**2.56**	up
8159554	UAP1L1	**2.55**	up
7972805	RAB20	**2.53**	up
8018754	CYGB	**2.53**	up
8005765	WSB1	**2.51**	up
7952526	CDON	**2.51**	up
8083063	SLC25A36	**2.49**	up
8007931	ITGB3	**2.49**	up
8025402	ANGPTL4	**2.48**	up
8120992	ZNF292	**2.48**	up

Affymetrix ID	Gene Symbol	Ni vs control	Regulation Ni
7986687	WHDC1L1	**2.48**	up
8104568	UNQ1870	**2.47**	up
8086799	CSPG5	**2.47**	up
7979179	ERO1L	**2.45**	up
8088958	GBE1	**2.44**	up
8089606	WDR52	**2.44**	up
8171653	MAP3K15	**2.42**	up
7995362	GPT2	**2.42**	up
8110158	ARL10	**2.40**	up
8054054	ANKRD36B	**2.40**	up
7948229	SLC43A3	**2.39**	up
8149749	TNFRSF10D	**2.37**	up
8004331	KCTD11	**2.34**	up
8120880	TPBG	**2.33**	up
7938390	ADM	**2.31**	up
8054064	ANKRD36B	**2.30**	up
8078529	STAC	**2.30**	up
8175023	ZDHHC9	**2.30**	up
7913858	PAQR7	**2.30**	up
7931951	SFMBT2	**2.30**	up
8015868	MPP2	**2.28**	up
7925250	GNG4	**2.27**	up
7993807	TMEM159	**2.26**	up
7978544	EGLN3	**2.26**	up
8027938	UPK1A	**2.25**	up
7928395	FUT11	**2.25**	up
8112592	FOXD1	**2.24**	up
8037430	ZNF404	**2.24**	up
8060344	TRIB3	**2.23**	up
7999614	NPIP	**2.22**	up
8115210	TNIP1	**2.22**	up
7962579	AMIGO2	**2.21**	up
7981514	AHNAK2	**2.20**	up
8011713	CXCL16	**2.20**	up
8096385	HERC3	**2.20**	up
7921099	CRABP2	**2.20**	up
8088642	LRIG1	**2.19**	up
8139488	IGFBP3	**2.19**	up
7989501	CA12	**2.19**	up
8093258	IQCG	**2.19**	up
8068022	MIRN155	**2.19**	up
8113073	ARRDC3	**2.19**	up
8005475	TRIM16L	**2.18**	up
8113214	GLRX	**2.18**	up
8070557	ZNF295	**2.18**	up
8035177	SLC35E1	**2.18**	up
8162276	NFIL3	**2.17**	up
7942674	TSKU	**2.17**	up
7984922	MPI	**2.17**	up
8072009	CRYBB2P1	**2.17**	up
8043697	ANKRD36B|FLJ40	**2.17**	up
7960143	ZNF84	**2.16**	up
8070819	PTTG1IP	**2.16**	up

Affymetrix ID	Gene Symbol	Ni vs control	Regulation Ni
7912520	NPPB	**2.16**	up
7905918	EFNA3	**2.16**	up
8084232	YEATS2	**2.15**	up
7938183	ZNF215	**2.15**	up
7930398	MXI1	**2.15**	up
7960744	C1R	**2.14**	up
7985016	SNX33	**2.14**	up
8168438	UPRT	**2.13**	up
7956301	LRP1	**2.13**	up
7984524	PAQR5	**2.12**	up
8003611	FAM57A	**2.12**	up
7997839	CDT1	**2.11**	up
8109120	AFAP1L1	**2.11**	up
8073007	MAFF	**2.10**	up
8093104	TM4SF19	**2.10**	up
8164810	RALGDS	**2.10**	up
8066431	ADA	**2.10**	up
7909545	TRAF5	**2.10**	up
8029580	RELB	**2.10**	up
7981943	PAR5	**2.10**	up
7962327	SLC2A13	**2.10**	up
7964872	PTPRB	**2.09**	up
7913593	TCEA3	**2.09**	up
8018793	JMJD6	**2.09**	up
8172425	SLC38A5	**2.08**	up
8149986	ZNF395|FBXO16	**2.07**	up
8065920	FER1L4	**2.07**	up
8008627	NOG	**2.05**	up
8013022	C17orf76	**2.05**	up
8010770	SLC16A3	**2.05**	up
8098604	ANKRD37	**2.05**	up
8068024	JAM2	**2.05**	up
7979663	RAB15	**2.05**	up
8144378	AGPAT5	**2.05**	up
7953603	C1S	**2.05**	up
7930074	NFKB2	**2.04**	up
8145454	BNIP3L	**2.04**	up
8107133	PAM	**2.04**	up
7955142	CACNB3	**2.04**	up
8074227	CECR5	**2.03**	up
7993624	SYT17	**2.03**	up
8160332	MLLT3	**2.02**	up
8049448	CENTG2	**2.02**	up
8143270	ZC3HAV1L	**2.01**	up
7933084	NAMPT	**2.01**	up
8129953	HIVEP2	**2.01**	up
7980485	DIO2	**6.27**	down
8173732	TAF9B	**5.35**	down
8111941	HMGCS1	**4.88**	down
7961142	OLR1	**4.67**	down
8140650	SEMA3E	**3.92**	down
8124196	DCDC2	**3.24**	down
8019392	FASN	**3.02**	down

Affymetrix ID	Gene Symbol	Ni vs control	Regulation Ni
8129458	ARHGAP18	**2.83**	down
8006433	CCL2	**2.81**	down
8098195	SC4MOL	**2.78**	down
8144669	FDFT1	**2.77**	down
7951662	CRYAB	**2.68**	down
8025828	LDLR	**2.67**	down
8106280	HMGCR	**2.66**	down
8138888	PDE1C	**2.60**	down
8108697	PCDHB5	**2.60**	down
8124380	HIST1H1A	**2.56**	down
8069689	ADAMTS5	**2.52**	down
8081657	CD200	**2.50**	down
7916584	TACSTD2	**2.48**	down
7904843	PDZK1	**2.43**	down
7931754	IDI1	**2.40**	down
7906900	DDR2	**2.39**	down
7954104	ATF7IP	**2.37**	down
7983650	SLC27A2	**2.34**	down
7934979	ANKRD1	**2.34**	down
8102342	ELOVL6	**2.32**	down
7962559	SLC38A4	**2.31**	down
8058552	IDH1	**2.27**	down
8154245	PDCD1LG2	**2.27**	down
8121734	ASF1A	**2.23**	down
8174201	BEX1	**2.22**	down
7947512	DKFZP586H2123	**2.21**	down
8045835	GALNT5	**2.21**	down
7915787	PIK3R3	**2.20**	down
8140686	SEMA3D	**2.20**	down
8051583	CYP1B1	**2.19**	down
8128383	COQ3	**2.17**	down
8135218	LRRC17	**2.16**	down
7902495	NEXN	**2.15**	down
7919591	GCUD2	**2.15**	down
8062041	ACSS2	**2.14**	down
7979241	BMP4	**2.14**	down
8023497	ATP8B1	**2.14**	down
7932584	PRTFDC1	**2.13**	down
8175052	AIFM1	**2.13**	down
8111772	DAB2	**2.12**	down
7916432	DHCR24	**2.11**	down
8128606	RTN4IP1	**2.10**	down
8037272	PSG7	**2.10**	down
8109597	UBLCP1	**2.09**	down
7961365	MANSC1	**2.09**	down
7962375	PRICKLE1	**2.08**	down
7965200	CCDC59	**2.08**	down
8106193	UTP15	**2.07**	down
8140668	SEMA3A	**2.07**	down
8151296	LACTB2	**2.07**	down
7958565	MVK	**2.06**	down
8148280	SQLE	**2.05**	down
7968872	DNAJC15	**2.05**	down

Affymetrix ID	Gene Symbol	Ni vs control	Regulation Ni
7925480	FH	**2.04**	down
7905986	FDPS	**2.02**	down
8067140	CYP24A1	**2.02**	down
8170590	NSDHL	**2.02**	down
7914557	SYNC1	**2.02**	down
8100347	SCFD2	**2.01**	down
8091411	TM4SF1	**2.00**	down
8008588	HLF	**2.00**	down
